# *Helicobacter pylori* Seropositivity in Patients with Interleukin-1 Polymorphisms Is Significantly Associated with ST-Segment Elevation Myocardial Infarction

**DOI:** 10.1371/journal.pone.0166240

**Published:** 2016-11-10

**Authors:** Noriaki Tabata, Daisuke Sueta, Tomonori Akasaka, Yuichiro Arima, Kenji Sakamoto, Eiichiro Yamamoto, Yasuhiro Izumiya, Megumi Yamamuro, Kenichi Tsujita, Sunao Kojima, Koichi Kaikita, Kazunori Morita, Kentaro Oniki, Junji Saruwatari, Kazuko Nakagawa, Seiji Hokimoto

**Affiliations:** 1 Department of Cardiovascular Medicine, Graduate School of Medical Sciences, Kumamoto University, Kumamoto City, Japan; 2 Division of Pharmacology and Therapeutics, Graduate School of Medical and Pharmaceutical Sciences, Kumamoto University, Kumamoto City, Japan; Institut Pasteur Paris, FRANCE

## Abstract

**Background:**

*Helicobacter pylori* infection and interleukin-1 polymorphisms are associated with an increased risk of gastric cancer. We examined the prevalence of *Helicobacter pylori* seropositivity and interleukin-1 polymorphisms between ST-segment elevation myocardial infarction and non-ST-segment elevation acute coronary syndrome patients.

**Methods:**

We recruited consecutive acute coronary syndrome patients, and 101 non-ST-segment elevation acute coronary syndrome patients and 103 ST-segment elevation myocardial infarction patients were enrolled. Interleukin-1 polymorphism analyses were performed for single nucleotide polymorphism in interleukin-1 beta-511 and the variable number of tandem repeats polymorphism in the interleukin-1 receptor antagonist by polymerase chain reaction. Immunoglobulin G antibodies against *Helicobacter pylori* and high sensitivity C-reactive protein were also measured.

**Results:**

The rates of the simultaneous presence of interleukin-1 polymorphisms and *Helicobacter pylori*-seropositivity between non-ST-segment elevation acute coronary syndrome and ST-segment elevation myocardial infarction groups were 25.7% and 42.7%, respectively (P = 0.012). *Helicobacter pylori*-seropositive subjects with interleukin-1 polymorphisms showed significantly higher levels of high sensitivity C-reactive protein (0.04–0.12 vs. 0.02–0.05; P<0.001). Multivariate logistic regression analysis revealed that the carriage of *Helicobacter pylori*-seropositivity and interleukin-1 polymorphisms was significantly associated with ST-segment elevation myocardial infarction (odds ratio, 2.32; 95% confidence interval, 1.23–4.37; P = 0.009). The C-statistic of conventional risk factors was 0.68 (P<0.001) and that including *Helicobacter pylori*-seropositivity and interleukin-1 polymorphisms was 0.70 (P<0.001); continuous net reclassification improvement was 34% (P = 0.0094) and integrated discrimination improvement was 3.0% (P = 0.014).

**Conclusions:**

The coincidence of *Helicobacter pylori*-seropositivity and interleukin-1 polymorphisms was significantly associated with higher levels of high sensitivity C-reactive protein and the increased risk of ST-segment elevation myocardial infarction.

## Introduction

Chronic bacterial infections have been suggested to be associated with the risk of acute coronary syndrome (ACS) [1,2]. *Helicobacter pylori* (*H*. *pylori*) is the most common chronic bacterial infection, and it has been demonstrated worldwide and in individuals of all ages. Previous studies have shown an association between *H*. *pylori* infection and coronary heart disease as well as acute coronary events [3,4], but other studies have not demonstrated such an association [5–7]. Some of *H*. *pylori* strains possess cytotoxin-associated gene-A (CagA), which is one of the major virulence factors of *H*. *pylori* and the high prevalence of CagA-positive *H*. *pylori* strain has been reported in Japan (94% among Japanese people) [8], and a current study showed a significant association between CagA seropositivity and myocardial infarction [9]. Thus, the investigation of the association of *H*. *pylori* seropositivity with atherosclerotic diseases might be important especially among Japanese people.

In the mechanism of *H*. *pylori*-induced diseases, host genetic factors in addition to bacterial and/or environmental factors determine the immune and inflammatory responses. The pro-inflammatory cytokine, interleukin (IL)-1 beta is an important candidate that influences the clinical outcomes of an *H*. *pylori* infection. The polymorphisms of IL-1 beta-511 genotype and IL-1 receptor antagonist (IL-1RN) are associated with a wide range of chronic inflammatory and autoimmune conditions [10–12]. A previous study reported that the carriage of IL-1 polymorphisms was significantly associated with *H*. *pylori*-related gastric inflammation, atrophy, and carcinogenesis [13], and we have recently found that *H*. *pylori*-infected patients with IL-1 polymorphisms were correlated with an increased level of inflammation and increased risk of myocardial infarction [14], and smoking patients with *H*. *pylori* infection and IL-1 polymorphisms had significantly increased risk of cardiovascular events after acute coronary syndrome (ACS) [15].

ST-segment elevation myocardial infarction (STEMI) and non-ST-segment elevation acute coronary syndrome (NSTE-ACS) represent a life-threatening condition that requires acute treatment [16,17], and patients with STEMI have a higher rate of in-hospital adverse cardiovascular events and in-hospital mortality [18]. Previous studies reported that clinical characteristics of STEMI and NSTE-ACS patients were significantly different; patients with STEMI patients were younger and had lower risk factors such as diabetes, hypertension, and dyslipidemia than NSTE-ACS patients [19,20]. The etiology of this difference remains unclear, but in the past, many papers have focused on the role of inflammation in the pathogenesis of ACS [21]. Moreover, it was reported that the inflammatory background was more significant in STEMI patients than in NSTE-ACS patients [22].

In the present study, we examined the prevalence of *H*. *pylori* infection and IL-1 polymorphisms between STEMI and NSTE-ACS patients.

## Materials and Methods

Consecutive ACS patients were recruited between January 2009 and December 2013 from Kumamoto University Hospital. Research cardiologists recorded the patients’ sociodemographic variables and medical history during their hospital stay. Information was obtained from the hospital medical records and by direct interviews with the patients, the family members, and treating physicians. We excluded patients with collagen diseases, other inflammatory diseases, severe liver and renal dysfunction, malignant diseases, and other severe co-morbidities. We also excluded patients who had already received *H*. *pylori* eradication treatments. The study complied with the Declaration of Helsinki, and the human ethics committee of Kumamoto University approved it. Written informed consents were obtained from all the patients.

All subjects provided venous blood samples for serology and genotyping at the time of cardiac catheterization during hospitalization for ACS. Blood samples for the serums were centrifuged, and the serums were stored at -80°C until analysis.

Immunoglobulin G (IgG) antibodies against *H*. *pylori* were measured using a direct enzyme-linked immunosorbent assay kit (E Plate Eiken *H*. *pylori* Antibody, Eiken Chemical Co., Ltd., Tokyo, Japan). All the measurements were performed at the Department of Cardiovascular Medicine, Kumamoto University in Japan. Levels of IgG were categorized as seropositive and seronegative for *H*. *pylori* according to a selective cutoff value (492 nm). Using the same kit, it was reported that the sensitivity and specificity of the kit with respect to cell culture and rapid urease test in 70 Japanese subjects were 100% and 80.0%, respectively [23]. The measurements of high sensitivity C-reactive protein (hs-CRP) level were performed in the laboratory of our hospital using routine enzymatic methods. Since acute phase proteins such as hs-CRP are up-regulated in MI patients [24], we collected data of hs-CRP 6–9 months after admission for ACS as far as possible, though medications such as statins subscribed on the admission might influence the CRP levels.

Genomic deoxyribonucleic acid (DNA) was extracted from whole blood using the DNA extractor WB kit (Wako Pure Chemical Industries, Ltd., Osaka, Japan) as described by Richards et al [25]. The IL-1 beta gene has three diallelic polymorphisms at positions -511, -31, and +3954 base pairs (bp) from the transcriptional start site [10], and there are data regarding the functional effects of these polymorphisms on IL-1-beta production [11,12]. A previous study reported that within the IL-1 beta gene, the T and C alleles at the -511 locus were in near total linkage disequilibrium with the C and T alleles at the -31 locus [26]. Another polymorphism at position +3954 in exon 5 has also been reported; however, the frequency of the mutant allele is very rare in Japanese populations, making it useless [26]. Therefore, we restricted the analysis to the IL-1 beta-511 locus. A single bp polymorphism at -511 in the promoter region of the IL-1 beta (rs16944) was determined using a real-time TaqMan allelic discrimination assay (Step One Plus Real-Time PCR system, version 2.1; Applied Biosystems, Tokyo, Japan) according to the protocols provided by the manufacturer (assay no.: C_1839943_10). The gene for the IL-1RN has a variable number of identical tandem repeats of 86 bp in length in intron 2, and the analysis of the IL-1RN polymorphism was performed as described previously [27]. IL-1RN alleles were stratified into two alleles according to the number of repeats: *2/*2, *2/L, and L/L (*2, the short allele of two repeats; L, the long allele of three or more repeats), and the less common allele 2 (IL-1RN*2) is associated with a wide range of chronic inflammatory and autoimmune conditions [10]. In this study, we defined IL-1 polymorphisms as the carriage of either IL-1 beta-511 T allele (C/T and T/T) or IL-1RN *2 allele.

We performed sample power analysis, and our pilot data indicated that the probability of the simultaneous presence of *H*. *pylori* infection and IL-1 polymorphisms among controls is 0.2. If the true probability of that among STEMI patients is 0.4, we needed to study at least 81 STEMI and 81 NSTE-ACS subjects in order to reject the null hypothesis that the exposure rates are equal with a probability power of 0.8. The Shapiro-Wilk test was used to assess the normal distribution of continuous data. Continuous variables with a normal distribution are expressed as the means ± standard deviations, whereas those with skewed distributions were expressed as the median value with interquartile range. Categorical data are presented as numbers or percentages. The Hardy-Weinberg equilibrium of alleles at individual loci was assessed using the chi-square test. The odds ratio (OR) and 95% confidence intervals (CI) were used to assess the risk of STEMI and were estimated by using logistic regression analysis. Predictors identified through univariate analysis, and other variables considered likely to have important prognostic value, were tested in a multivariate analysis. Estimates of the C-statistic for predictors were calculated before and after the addition of the simultaneous presence of *H*. *pylori* and IL-1 polymorphisms; its incremental effect for predicting STEMI was evaluated using the net reclassification index (NRI) and integrated discrimination index (IDI) as previously described [28]. A P value <0.05 was considered to denote the presence of a statistically significant difference. Statistical analyses were performed using SPSS, version 22 (IBM Corp., Armonk, NY, USA).

## Results

**Fig 1:**
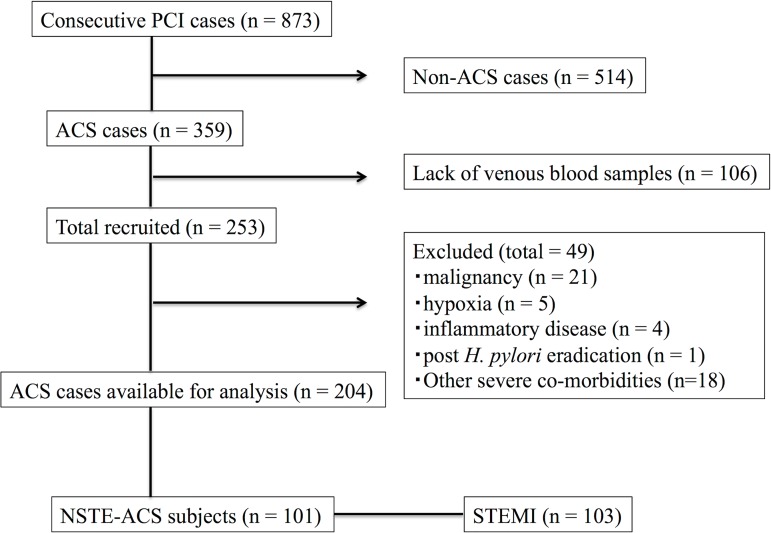
Study flow chart of the present study. From consecutive 873 PCI cases, we excluded non-ACS cases (n = 514), cases without venous blood samples (n = 106), and patients with exclusion criteria (n = 49). Finally we analyzed available 204 ACS cases. PCI, percutaneous coronary intervention; ACS, acute coronary syndrome; *H*. *pylori*, *Helicobacter pylori*; NSTE-ACS, non-ST-segment elevation acute coronary syndrome; STEMI, ST-segment elevation myocardial infarction.

Consecutive 204 patients (101 NSTE-ACS patients and 103 STEMI patients) were enrolled in this study (Fig 1). Table 1 compares the clinical characteristics between the NSTE-ACS and STEMI groups. Male sex and current smoker were significantly higher (60.4% versus 75.7%, P = 0.024, and 13.9% versus 35.0%, P = 0.001, respectively) in the STEMI group compared with the NSTE-ACS group. There was no difference in the rates of patients who have past (5.0% versus 6.8%; P = 0.768) or current (5.0% versus 1.9%; P = 0.277) medical history of gastroduodenal diseases (gastritis or peptic ulcer) and the rates of patients who had been prescribed proton-pump inhibitor (15.8% and 13.6%; P = 0.696) between NSTE-ACS and STEMI, respectively. The prevalence of *H*. *pylori* IgG antibody in NSTE-ACS and STEMI groups were 36.6% and 51.5%, respectively (P = 0.036).

**Table 1:** Clinical characteristics between NSTE-ACS and STEMI groups.

	**Total (n = 204)**	**NSTE-ACS (n = 101)**	**STEMI (n = 103)**	***P* value**
Male (%)	139 (68.1)	61 (60.4)	78 (75.7)	0.024
Age (years)	69.6 ± 11.0	70.0 ± 10.7	69.1 ± 11.2	0.553
BMI (kg/m^2^)	23.8 ± 3.7	23.8 ± 3.9	23.7 ± 3.4	0.891
AC (cm)	87.5 ± 9.1	86.8 ± 9.5	88.1 ± 8.7	0.335
Diabetes (%)	86 (42.2)	42 (41.6)	44 (42.7)	0.888
Hypertension (%)	154 (75.5)	81 (80.2)	73 (70.9)	0.144
Dyslipidemia (%)	149 (73.0)	77 (76.2)	72 (69.9)	0.346
Current smoking (%)	50 (24.5)	14 (13.9)	36 (35.0)	0.001
Family history (%)	53 (26.0)	30 (29.7)	23 (22.3)	0.265
CKD (%)	62 (30.4)	29 (28.7)	33 (32.0)	0.65
Previous MI (%)	8 (3.9)	2 (2.0)	6 (5.8)	0.279
Previous stroke (%)	19 (9.3)	8 (7.9)	11 (10.7)	0.631
PAD (%)	15 (7.4)	8 (7.9)	7 (6.8)	0.795
Past GD diseases	12 (5.9)	5 (5.0)	7 (6.8)	0.768
Current GD diseases	7 (3.4)	5 (5.0)	2 (1.9)	0.277
Proton-pump inhibitors	30 (14.7)	16 (15.8)	14 (13.6)	0.696
hs-CRP (mg/dl)	0.04 (0.02–0.07)	0.04 (0.02–0.06)	0.04 (0.02–0.08)	0.901
*H*. *pylori* IgG (%)	90 (44.1)	37 (36.6)	53 (51.5)	0.036
IL-1 beta-511 C/C (%)	60 (29.7)	34 (33.7)	26 (25.7)	0.281
IL-1 beta-511 C/T (%)	88 (43.6)	38 (37.6)	50 (49.5)	0.118
IL-1 beta-511 T/T (%)	54 (26.7)	29 (28.7)	25 (24.8)	0.634
IL-1RN *2 allele (%)	27 (13.5)	16 (16.2)	11 (10.9)	0.306
IL-1 polymorphisms	146 (72.3)	70 (69.3)	76 (75.2)	0.432

Abbreviations: NSTE-ACS, Non ST segment elevation acute coronary syndrome; STEMI, ST segment elevation acute myocardial infarction; BMI, body mass index; AC, abdominal circumference; CKD, chronic kidney disease; MI, myocardial infarction; PAD, peripheral arterial disease; GD, gastroduodenal; hs-CRP, high-sensitivity C-reactive protein; *H*. *pylori*, *Helicobacter pylori*; IgG, immunoglobulin; IL-1, interleukin-1; IL-1RN, interleukin-1 antagonist

The genotype frequencies of the IL-1 beta-511 C/C, C/T, and T/T and IL-1RN between NSTE-ACS and STEMI groups were shown in Table 1. The genotype frequencies of the IL-1 beta-511 C/C, C/T, and T/T were 33.7%, 37.6%, and 28.7%, respectively in the NSTE-ACS group, and 25.7%, 49.5%, and 24.8%, respectively, in the STEMI group. The distribution of the IL-1RN L/L, *2/L, and *2/*2 were 83.8%, 15.2%, and 1%, respectively, in the NSTE-ACS group, and 89.1%, 10.9%, and 0%, respectively, in the STEMI group. The genotypes of the IL-1 and the alleles of the IL-1RN were in Hardy-Weinberg equilibrium (P = 0.23 and P = 0.48, respectively). IL-1 polymorphisms between NSTE-ACS and STEMI groups were 69.3% and 75.2% (P = 0.432). The prevalence of STEMI in patients with and without *H*. *pylori* seropositivity was 62.3% and 42.9% in patients with IL-1 polymorphisms (P = 0.021); on the other hand, that was 45.0% and 44.4% (P = 1.0), respectively.

**Fig 2:**
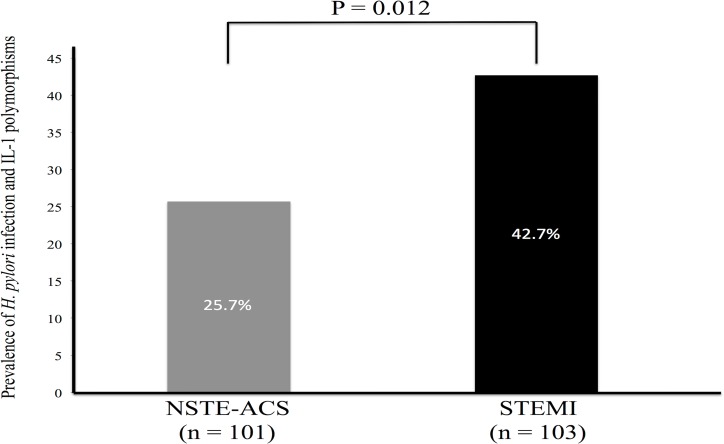
Prevalence of the coincidence of *Helicobacter pylori* seropositivity and interleukin-1 polymorphisms between NSTE-ACS and STEMI groups. NSTE-ACS, non-ST-segment elevation acute coronary syndrome; STEMI, ST-segment elevation myocardial infarction.

**Fig 3:**
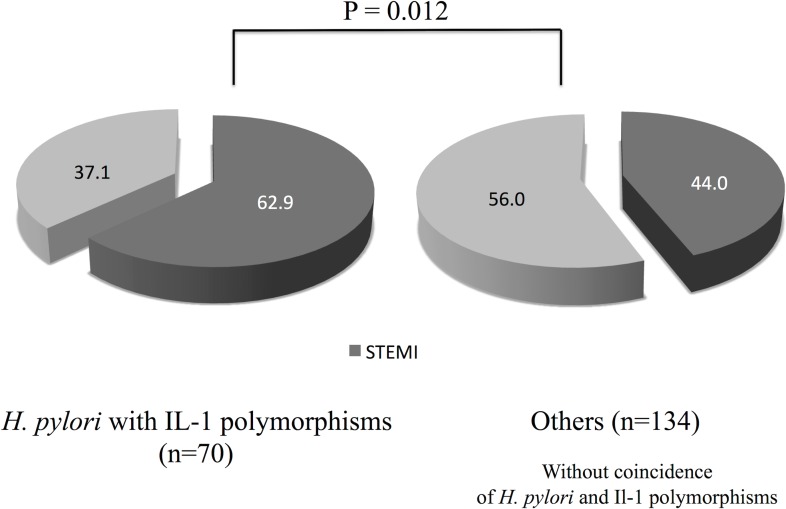
Prevalence of STEMI according to the coincidence of *Helicobacter pylori* seropositivity and interleukin-1 polymorphisms. STEMI, ST-segment elevation myocardial infarction.

**Fig 4:**
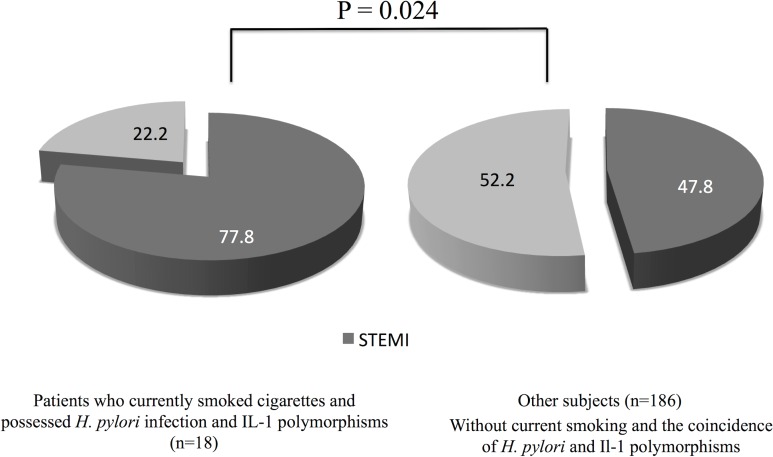
Prevalence of STEMI according to the current smoke exposure and the coincidence of *Helicobacter pylori* seropositivity and interleukin-1 polymorphisms. STEMI, ST-segment elevation myocardial infarction.

The rates of the simultaneous presence of IL-1 polymorphisms and *H*. *pylori* infection between NSTE-ACS and STEMI groups were 25.7% and 42.7%, respectively (P = 0.012) (Fig 2). Table 2 shows the clinical characteristics according to the simultaneous prevalence of *H*. *pylori* seropositivity and IL-1 polymorphisms. We found significantly higher rate of STEMI (Fig 3) and higher serum levels of hs-CRP in patients having both of the *H*. *pylori* seropositivity and IL-1 polymorphisms. We performed logistic regression analysis for STEMI (Tables 3 and 4). Multivariate analysis revealed that the coincidence of *H*. *pylori* infection and IL-1 polymorphisms (OR, 2.32; 95% CI, 1.23–4.37; P = 0.009) and current smoking (OR, 3.44; 95% CI, 1.58–7.52; P = 0.002) were significantly associated with STEMI after adjusting for age, male sex, diabetes, hypertension, dyslipidemia, obesity, current smoking, and family history. In patients who currently smoked cigarettes and possessed *H*. *pylori* infection and IL-1 polymorphisms (N = 18), 14 subjects (77.8%) were found to be STEMI (Fig 4).

**Table 2:** Clinical characteristics according to simultaneous presence of *H*. *pylori* and IL-1 polymorphisms.

	***H*. *pylori* with IL-1 polymorphisms (n = 70)**	**Others (n = 134) (Without coincidence of *H*. *pylori* and IL-1 polymorphisms)**	***P* value**
Male (%)	51 (72.9)	88 (65.7)	0.344
Age (years)	68.7 ± 9.8	70.0 ± 11.5	0.414
BMI (kg/m^2^)	24.1 ± 3.9	23.6 ± 3.6	0.426
Abdominal Circumference (cm)	87.5 ± 9.4	87.4 ± 8.9	0.968
Diabetes (%)	32 (45.7)	54 (40.3)	0.460
Hypertension (%)	55 (78.6)	99 (73.9)	0.497
Dyslipidemia (%)	50 (71.4)	99 (73.9)	0.741
Current smoking (%)	18 (25.7)	32 (23.9)	0.864
Family history (%)	21 (30.0)	32 (23.9)	0.401
CKD (%)	17 (24.3)	45 (33.6)	0.201
Previous MI (%)	3 (4.3)	5 (3.7)	1.0
Previous stroke (%)	7 (10.0)	12 (9.0)	0.804
PAD (%)	5 (7.1)	10 (7.5)	1.0
hs-CRP (mg/dl)	0.06 (0.04–0.12)	0.03 (0.02–0.05)	< 0.001
STEMI (%)	44 (62.9)	59 (44.0)	0.012

Abbreviations: *H*. *pylori*, *Helicobacter pylori*; IL-1, interleukin-1; BMI, body mass index; CKD, chronic kidney disease; MI, myocardial infarction; PAD, peripheral arterial disease; hs-CRP, high-sensitivity C-reactive protein; STEMI, ST-segment elevation myocardial infarction

**Table 3:** Logistic regression analysis for STEMI.

				Multivariate Regression		
Variable	Univariate Regression			Model 1		
	OR	95% CI	P value	OR	95% CI	P value
*HP* seropositivity	1.83	1.05–3.21	0.034	1.71	0.94–3.10	0.077
IL-1 polymorphisms	1.35	0.73–2.50	0.35			
*HP* with IL-1 polymorphisms	2.15	1.19–3.89	0.011			
Age	0.99	0.97–1.02	0.55	1.00	0.97–1.04	0.79
Male sex	2.05	1.12–3.73	0.02	1.54	0.80–2.97	0.20
Diabetes	1.05	0.60–1.83	0.87	1.21	0.65–2.26	0.54
Hypertension	0.60	0.31–1.15	0.12	0.54	0.27–1.08	0.082
Dyslipidemia	0.72	0.39–1.35	0.31	0.63	0.31–1.28	0.20
Obesity	1.02	0.57–1.82	0.96	1.24	0.64–2.41	0.52
Current smoking	3.34	1.67–6.69	0.001	3.24	1.50–7.0	0.003
Family history	0.68	0.36–1.28	0.23	0.63	0.31–1.29	0.20
Previous MI	3.06	0.60–15.5	0.18			
Previous stroke	1.39	0.54–3.61	0.50			
PAD	0.85	0.30–2.43	0.76			
CKD	1.17	0.64–2.13	0.61			

Abbreviations: OR, odds ratio; CI, confidence interval; STEMI, ST-segment elevation myocardial infarction; *HP*, *Helicobacter pylori*; IL-1, interleukin-1; MI, myocardial infarction; PAD, peripheral arterial disease; CKD, chronic kidney disease

**Table 4:** Logistic regression analysis for STEMI.

	Multivariate Regression					
	Model 2			Model 3		
Variable	OR	95% CI	P value	OR	95% CI	P value
*HP* seropositivity						
IL-1 polymorphisms	1.60	0.83–3.11	0.16			
*HP* with IL-1 polymorphisms				2.32	1.23–4.37	0.009
Age	1.01	0.97–1.04	0.73	1.01	0.97–1.04	0.75
Male sex	1.56	0.81–2.99	0.19	1.56	0.80–3.02	0.19
Diabetes	1.25	0.67–2.33	0.48	1.23	0.66–2.31	0.52
Hypertension	0.56	0.27–1.13	0.10	0.51	0.25–1.05	0.066
Dyslipidemia	0.56	0.27–1.14	0.11	0.60	0.29–1.24	0.17
Obesity	1.26	0.65–2.45	0.50	1.25	0.64–2.44	0.52
Current smoking	3.50	1.60–7.66	0.002	3.44	1.58–7.52	0.002
Family history	0.62	0.31–1.28	0.20	0.60	0.29–1.24	0.17

Abbreviations: OR, odds ratio; CI, confidence interval; STEMI, ST-segment elevation myocardial infarction; *HP*, *Helicobacter pylori*; IL-1, interleukin-1

The C-statistic of the conventional risk factors (age, male sex, diabetes, hypertension, dyslipidemia, obesity, current smoking, and family history) was 0.68 (95% CI, 0.60–0.75; P<0.001) and that after adding *H*. *pylori* infection and IL-1 polymorphisms was 0.70 (95% CI, 0.63–0.77; P<0.001). We reclassified conventional risk factors by including *H*. *pylori* infection and IL-1 polymorphisms, and the continuous NRI was 34% (95% CI, 8–60%; P = 0.0094) and IDI was 3.0% (0.62–5.4%; P = 0.014) (Table 5).

**Table 5:** Comparison of Risk Prediction Models before and after adding *H*. *pylori* and IL-1 polymorphisms.

	CRFs	CRFs + *H*. *pylori* + IL-1 polymorphisms
C-statistic	0.68 (0.60–0.75)	0.70 (0.63–0.77)
Continuous NRI	34% (8.0–60%; P = 0.0094)	
IDI	3.0% (0.62–5.4%; P = 0.014)	

Abbreviations: *H*. *pylori*, *Helicobacter pylori*; IL-1, interleukin-1; CRFs, conventional risk factors; NRI, net reclassification improvement; IDI, integrated discrimination improvement

## Discussion

This is the first study to examine the association of *H*. *pylori* infection and IL-1 polymorphisms with ACS patients. The present study has three important findings. First, *H*. *pylori* seropositivity and IL-1 polymorphisms were more significantly found in STEMI patients than NSTE-ACS patients. Second, patients with *H*. *pylori* infection and IL-1 polymorphisms showed significantly higher levels of hs-CRP than patients without them. Third, the coincidence of *H*. *pylori* infection and IL-1 polymorphisms and current smoke exposure were significant and independent predictors of the incidence of STEMI.

STEMI represents a more life-threatening condition and requires acute treatment, and patients with STEMI have a higher rate of in-hospital adverse cardiovascular events and in-hospital mortality, compared to NSTE-ACS [18]. Therefore, identifying the risk factor of STEMI is clinically of great significance. The important mechanism of ACS is a rupture of a vulnerable plaque and subsequent thrombus formation. In the previous intravascular ultrasound studies, it was reported that plaque characteristics of the culprit lesion with STEMI had greater plaque burden and more markers of plaque vulnerability than NSTE-ACS patients [29,30]. In contrast, it has been reported that patients presenting with STEMI were younger and had fewer risk factors of atherosclerosis, which seems paradoxical. Inflammatory background is more significant in the pathogenesis of STEMI than that of NSTE-ACS [22], so chronic inflammation might have the key role to this etiological difference. Atherosclerosis is an inflammatory disease, and inflammation has a fundamental role in all the stages of atherosclerosis from initiation through progression and, finally, in the thrombotic complications of atherosclerosis.

The relationship between *H*. *pylori* infection and atherosclerosis has been detected already [31], and a recent meta-analysis suggested a relationship between *H*. *pylori* infection and the risk of myocardial infarction [32]. Previous studies have demonstrated that *H*. *pylori* infection might cause vulnerable plaques [33]. Some of *H*. *pylori* strains possess cytotoxin-associated gene-A (CagA), which is one of the major virulence factors of *H*. *pylori* [34,35], and high prevalence of CagA-positive *H*. *pylori* strain has been reported in Japan [8]. Recently, it was reported that exosomes secreted from CagA-expressing gastric epithelial cells might enter into systemic circulation, leading to distant organs and tissues, and CagA-containing exosomes might be involved in the pathogenicity of extragastric disorders [36]. Another study suggested the association of CagA seropositivity with atherosclerotic plaque instability [37]. Thus, it is rationale that *H*. *pylori* seropositivity especially in Japanese people might be correlated with acute coronary events possibly by the mechanism of CagA-containing exosomes.

Several studies stressed the role of cytokine polymorphisms in modulating the levels of inflammation associated with chronic low-grade infections such as periodontal infections, Chlamydia pneumonia, and *H*. *pylori* infection, leading to an increased risk of atherosclerosis [38,39]. In the present study, we did not demonstrate the local levels of IL-1 beta, but IL-1 genetic polymorphisms influence *H*. *pylori*-related gastric mucosal IL-1 beta levels [13]. Thus, the IL-1 polymorphisms could be important determinants of the inflammatory response in patients with *H*. *pylori* infection, because cytokines such as IL-1 beta is important in the inhibition of gastric acid secretion [40], and the inhibition of gastric acid enables larger colonization of *H*. *pylori* bacteria [41], leading to continuous chronic infection of *H*. *pylori*. The carriage of IL-1 polymorphisms is significantly associated with *H*. *pylori*-related gastric cancer, but the association of the coincidence of *H*. *pylori* infection and IL-1 polymorphisms with acute coronary events has not been reported. Our results suggested that there is a possibility that *H*. *pylori* infection and IL-1 polymorphisms might cause a synergistic effect on the incidence of cardiovascular events.

Because there are insufficient reports about the association of *H*. *pylori* infection, IL-1 polymorphisms, and ACS pathogenesis, it is unclear why the coincidence of *H*. *pylori* infection and IL-1 polymorphisms could be more associated with STEMI than NSTE-ACS. The blood clot properties and resistance to lysis are determined by various factors including genetic, clinical, and environmental factors including acute cigarette smoke exposure [42], and growing evidence indicated that altered plasma fibrin properties of reduced clot permeability and impaired fibrinolysis contribute to the progressive of atherosclerotic vascular disease and the occurrence of thrombotic manifestations [43,44]. It was recently reported that clots of dense networks in ACS patients were correlated with raised inflammation [45]. Considering previous reports, there is a possibility that the coincidence of *H*. *pylori* infection and IL-1 genetic factors is more associated with inflammation reflected by raised CRP and thrombotic properties of reduced permeability and fibrinolysis, resulting in more incidence of STEMI.

In the present study, we evaluated the added predictive ability of *H*. *pylori* seropositivity along with IL-1 polymorphisms for STEMI by comparing the model of conventional risk factors with that including *H*. *pylori* seropositivity and IL-1 polymorphisms. Though the increase of C-statistic was not much, adding these factors improved the prediction of STEMI by two current statistical methods of NRI and IDI; therefore, simultaneous evaluation of environmental and genetic factor might improve the risk stratification of STEMI beyond conventional risk factors. The present study provided the possibility that *H*. *pylori* infection with IL-1 polymorphisms might be a precipitating factor of STEMI, and these conditions might cause the differential onset of ACS. Our results suggested that evaluating both the *H*. *pylori* infection and IL-1 polymorphisms would improve the risk stratification of the incidence of STEMI, and there is a possibility that the eradication of *H*. *pylori* might decrease the risk of life-threatening STEMI.

The present study has several limitations. First, this study is that it was performed at a single center, and the number of study subjects was small, so the small size of the study limits the results to merely association, without implying causation. Actually, the association between coincidence of IL-1 polymorphisms plus *H*. *pylori* infection and STEMI presentation does not prove causation. Second, the frequencies of IL-1 beta-511 genotype and IL-1RN *2 allele are different among ethnic populations, and our results may not be applicable all over the world. Moreover, we did not demonstrate local levels of IL-1 beta, and did not measure the serum levels of inflammatory cytokines such as IL-1 beta, IL-6, and tumor necrosis factor; thus, we did not demonstrate that the inflammation was induced by the elevation of inflammatory cytokine levels. In addition, we estimated the prevalence of *H*. *pylori* infection only by the results of IgG serology, and we did not measure immunoglobulin (IgM) serology. Thus, we could not assess new infections or disease activities. And we did not study several other organisms such as Chlamydia pneumonia, herpes simplex virus, and cytomegalovirus, and we cannot exclude the possibility that the aggregate burden of chronic infections, rather than single organism, contributed to the results of this study.

In conclusion, the coincidence of *H*. *pylori* infection and IL-1 polymorphisms was significantly associated with higher levels of hs-CRP and the increased risk of STEMI.
